# Therapeutical Usefulness of PD-1/PD-L1 Inhibitors in Aggressive or Metastatic Pituitary Tumours

**DOI:** 10.3390/cancers16173033

**Published:** 2024-08-30

**Authors:** Mariana Lopes-Pinto, Ema Lacerda-Nobre, Ana Luísa Silva, Pedro Marques

**Affiliations:** 1 Endocrinology Department, Unidade Local de Saúde de Santa Maria, Hospital de Santa Maria, 1649-035 Lisbon, Portugal; 2 Faculdade de Medicina, Universidade de Lisboa, 1649-028 Lisbon, Portugal; 3 Instituto de Saúde Ambiental, Faculdade de Medicina, Universidade de Lisboa, 1649-028 Lisbon, Portugal; 4 Faculdade de Medicina, Universidade Católica Portuguesa, 1649-023 Lisbon, Portugal; 5 Pituitary Tumor Unit, Endocrinology Department, Hospital CUF Descobertas, 1998-018 Lisbon, Portugal

**Keywords:** pituitary tumour, pituitary adenoma, immune checkpoint inhibitors, PD-1, PD-L1, immunotherapy, ipilimumab, nivolumab, pembrolizumab

## Abstract

**Simple Summary:**

Immune checkpoint inhibitors (ICIs) have been experimentally used in refractory pituitary neuroendocrine tumours (PitNETs). We reviewed the published data on PitNETs treated with PD-1/PD-L1 inhibitors. Demographics, clinical–pathological features, treatment details, radiological and biochemical responses, and survival were evaluated. Among twenty-nine ICI-treated PitNETs, eighteen secreted adrenocorticotropic hormone (ACTH) (62.1%), seven were prolactinomas (24.1%), and four were non-functioning PitNETs. All patients underwent various therapies prior to ICI treatment. A positive radiological response (i.e., partial/complete radiological response and stable disease) was observed in eighteen cases (62.1%), of which ten and four were ACTH- and prolactin-secreting PitNETs, respectively. Hormonal levels reduced or stabilised after using ICIs in 11 cases (64.7%). The median survival after using ICIs was 13 months. These data suggest a promising role of ICIs in patients with PitNETs refractory to other treatment modalities.

**Abstract:**

Therapeutic options for pituitary neuroendocrine tumours (PitNETs) refractory to temozolomide are scarce. Immune checkpoint inhibitors (ICIs), particularly inhibitors of the programmed cell death-1 (PD-1) pathway and its ligand (PD-L1), have been experimentally used in aggressive or metastatic PitNETs. We aimed to study the therapeutic usefulness of anti-PD-1 drugs in patients with aggressive or metastatic PitNETs. Published cases and case series involving patients with PitNETs treated with PD-1/PD-L1 inhibitors were reviewed. Demographic data, clinical–pathological features, previous therapies, drug dosage and posology, and the best radiological and biochemical responses, as well as survival data, were evaluated. We identified 29 cases of aggressive (*n* = 13) or metastatic (*n* = 16) PitNETs treated with PD-1/PD-L1 inhibitors. The hypersecretion of adrenocorticotropic hormone (ACTH) was documented in eighteen cases (62.1%), seven were prolactinomas (24.1%), and four were non-functioning PitNETs. All patients underwent various therapies prior to using ICIs. Overall, a positive radiological response (i.e., partial/complete radiological response and stable disease) was observed in eighteen of twenty-nine cases (62.1%), of which ten and four were ACTH- and prolactin-secreting PitNETs, respectively. Hormonal levels reduced or stabilised after using ICIs in 11 of the 17 functioning PitNET cases with available data (64.7%). The median survival of patients treated with ICIs was 13 months, with a maximum of 42 months in two ACTH-secreting tumours. Among 29 patients with PitNETs treated with PD-1/PD-L1 inhibitors, the positive radiological and biochemical response rates were 62.1% and 64.7%, respectively. Altogether, these data suggest a promising role of ICIs in patients with aggressive or metastatic PitNETs refractory to other treatment modalities.

## 1. Introduction

Pituitary neuroendocrine tumours (PitNETs) are usually benign neoplasms that arise from the anterior pituitary gland [1,2,3,4,5,6]. PitNETs typically grow slowly and can be treated by surgery and/or standard medical therapies while radiotherapy may be needed in some cases to arrest tumour growth. However, a small subset of PitNETs may present a progressive or recurrent growth not controlled by repeated surgery, radiotherapy, and/or medical therapy, being referred to as aggressive PitNETs [1,2,7,8,9,10].

Temozolomide has been used as first-line treatment for aggressive or metastatic PitNETs as it may lead to clinical benefits and radiological responses in up to 33% of cases and can increase survival rates in about 5 years [6,11]. However, not all PitNETs respond, and even those that show an initial response may become refractory to temozolomide over time [4,8,11,12].

The therapeutic arsenal in refractory PitNETs lacks effective options. Novel treatments such as those using tyrosine kinase inhibitors, multiple kinase inhibitors, mTOR inhibitors, bevacizumab, peptide receptor radionuclide therapy, and immune checkpoint inhibitors (ICIs) have been experimentally used, but robust data regarding their effectiveness and safety in patients with aggressive or metastatic PitNETs are lacking [3,4,13,14,15].

The use of ICIs in cancer relies on the immune recognition and destruction of malignant tumour cells by T cells [16,17,18,19]. The anti-programmed-cell-death-protein-1 (PD-1) monoclonal antibodies nivolumab and pembrolizumab promote tumour cell destruction by inhibiting the PD-1 ligand (PD-L1) expressed on tumour cells to be recognised as self by the immune system, while ipilimumab is a monoclonal antibody that blocks the cytotoxic T-lymphocyte-associated protein 4 (CTLA-4), a receptor on the T cell surface that inhibits the inappropriate or prolonged activation of T cells [16,17,20,21]. The interest in immunotherapy with ICIs in patients with aggressive or refractory PitNETs has emerged over recent years based on (i) the proven benefit of ICI treatment in other solid tumours, including renal cell carcinoma, non-small cell lung carcinoma, and melanoma [22,23,24,25]; (ii) the occurrence of hypophysitis secondary to ICIs, suggesting that the pituitary gland may be targetable with ICI, including for the pathways related with PD-1/PD-L1 or with CTLA-4 pathways [16,26]; and (iii) the fact that PD-L1 is expressed by PitNETs [27], which may transpire the biological relevance of immune-checkpoint-related pathways in pituitary tumour progression and invasiveness, as well as in the response to ICI treatment [4,18,19,24,26,27,28,29,30,31,32,33]. Anti-PD-1 drugs have been used off-label in patients with aggressive or metastatic PitNETs, in monotherapy, and in combination with other drugs or novel therapies, and reported individually or in small series of cases [11,34]. Here, we review, compile, and collectively analyse the published studies with PitNET patients who were treated with PD-1/PD-L1 pathway inhibitors.

## 2. Materials and Methods

### 2.1. Search Methodology and Selection of the Studies and Case Reports

We reviewed the literature concerning PitNET patients treated with anti-PD-1 drugs by undertaking a PubMed search using the following terms: “pituitary carcinoma”, “pituitary adenoma”, “pituitary tumour”, “pituitary neuroendocrine tumour”, “PitNET”, “PD-1”, “PD-L1”, “immune checkpoint inhibitors”, “immunotherapy”, “anti-PD-1”, “anti-PD-L1”, “nivolumab”, and “pembrolizumab”. The indexed manuscripts published in English up to May 2024 were reviewed and evaluated, including relevant articles from the reference lists of each publication.

### 2.2. Demographic, Clinicopathological, Biochemical, and Radiological Features

All case reports and small case series involving PitNET patients treated with PD-1/PD-L1 pathway inhibitors were thoroughly analysed.

The demographic data including sex, age at diagnosis of PitNET, and clinicopathological features were collected, including the PitNET subtype, absence or presence of metastasis (aggressive vs. metastatic PitNET, respectively), and PD-L1 expression status. Previous therapies to anti-PD-1 drugs were also assessed, as were treatment sequences in each case. Only cases which specified the anti-PD-1 drug used were considered and data regarding drug posology and number of cycles were collected when available. We also evaluated the best radiological and biochemical response obtained after anti-PD-1 treatment in each case. The radiological response was considered complete when there was total remission of the primary tumour, partial response when the tumour involuted but disease remained present, progressive when there was lack of response to treatment, stable when there was no clear response or progression, and dissociated response when there was an opposite effect of anti-PD-1 in the primary tumour and metastatic deposits. A positive radiological effect was considered in the case of partial/complete radiological or stable disease response.

The best biochemical response in functioning PitNETs was described as complete response when normal hormonal levels were achieved after starting anti-PD-1 treatment with or without other treatment modalities, partial response when hormone levels lowered but remained in excess, and stable when hormone secretion did not increase nor decrease after treatment. A positive biochemical effect of PD-1 inhibitors was assumed when hormone levels remained stable or decreased after treatment. Survival data, presented in terms of number of months after the start and end of anti-PD-1 drug regimen, were also reviewed when available and collectively analysed.

### 2.3. Statistical Analysis

Categorical variables are presented as absolute numbers and percentages while continuous variables with non-normal distribution are presented as medians.

Descriptive statistical analysis was performed using the SPSS software (version 26.0, IBM, New York, NY, USA).

## 3. Results

### 3.1. Demographics, Clinical Features, PD-L1 Expression Status, and Prior Treatments

**Figure 1:**
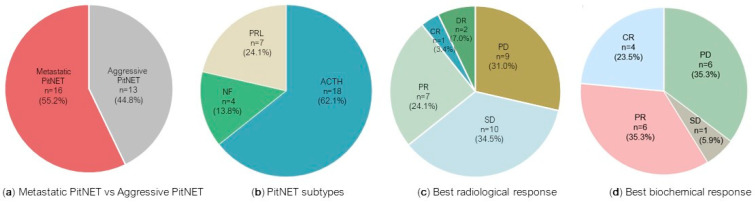
Stage, tumour subtypes, and response to treatment with PD-1 inhibitors in the cohort of 29 PitNETs. Data are presented regarding tumour stage as metastatic PitNET versus aggressive PitNET (**a**), PitNET subtypes (**b**), best radiological response (**c**), and best biochemical response (**d**). Results are presented as *n* (%). Regarding the best biochemical response (**d**), only 17 of the 25 functioning tumours had biochemical response data available. ACTH: adrenocorticotropic hormone-secreting PitNET; CR: complete response; DR: dissociated response (opposite effect of immune checkpoint inhibitors in primary tumour and metastases); NF: non-functioning PitNET; PD: progressive disease; PD-1: programmed cell death protein 1; PitNET: pituitary neuroendocrine tumour; PR: partial response; PRL: prolactin-secreting PitNET; SD: stable disease.

We identified 29 patients with aggressive PitNETs (*n* = 13) or metastatic PitNETs (*n* = 16) who had been treated with anti-PD-1 drugs in monotherapy or combined with other non-PD-1 ICIs (Table 1 and Figure 1a). Nine out of the twenty-nine patients were female (31%) and the median age at diagnosis was 43 years. Eighteen patients (62.1%) had an ACTH-secreting PitNET, ten of which were metastatic, and seven patients (24.1%) had a prolactinoma, three of which were metastatic; there were also one aggressive NF-PitNET and three metastatic NF-PitNET cases (Figure 1b).

The PD-L1 expression status was assessed in twenty-three of the twenty-nine PitNETs and it was positive in seven cases (30.4%): two NF-PitNETs (one metastatic), three prolactinomas (one metastatic), and two ACTH-secreting PitNETs [4,11,15,35,36]. All patients were submitted to neurosurgery, with an average of 2.5 operations (minimum: 1, maximum: 4). Different combinations of treatments were employed, including cabergoline, pasireotide, temozolomide, pazopanib, bevacizumab, radiotherapy, surgery and radiotherapy of metastasis, and peptide receptor radionuclide therapy (PRRT) (Table 1). Monotherapy with temozolomide was used as the systemic chemotherapy in 25 cases, with a median of nine cycles (minimum: three, maximum: forty-six). In one case, temozolomide was used in combination with capecitabine [37]. In the subgroup of 18 ACTH-secreting PitNETs, bilateral adrenalectomy was performed in nine cases (three aggressive and six metastatic PitNETs) (Table 1).

**Table 1:** Radiological and biochemical response to PD-1 inhibitors in a cohort of 29 published PitNET patients.

Case Report	Sex	Age at Diagnosis/Age at Anti-PD-1 Treatment (Years)	PitNET Subtype	PD-L1 Status	Previous Treatment	Anti-PD-1 Drug and Dose	Number of Cycles	Radiological Response	Biochemical Response	Survival after the Start/End of Anti-PD-1 Drug (Months)
Lin et al. [37], 2018 *J Clin Endocrinol Metab* (PMID: 30085142) Lin et al. [14], 2021 *J Endocr Soc* (PMID: 4466766)	F	35	ACTH-PitNET (metastatic)	-	NS (4x), RT (3x), PAS, KET, CAB/KET, MIF, MET, BA, CAPTEM (4 + 2 cycles), Carboplatin/Etoposide, MS, RT metastasis (2x), PRRT	IPI 3 mg/kg + NIVO 1 mg/kg 3/3 weeks IPI 3 mg/kg + NIVO 1 mg/kg 3/3 weeks NIVO 3 mg/kg 3/3 weeks IPI 3 mg/kg + NIVO 1 mg/kg 3/3 weeks	5 4 4	Partial response Partial response Dissociated response with partial response of metastasis and tumour growth	Partial response, followed by progression Stable Stable	42
Caccese et al. [38], 2020 *Anticancer Drugs* (PMID: 31702999)	M	47	ACTH-PitNET *	-	NS (3x), RT, PAS, TMZ (6cycles)	Pembrolizumab 200 mg	4	Progression	Progression	NA
Duhamel et al. [39], 2020 *J Pers Med* (PMID: 32823651)	M	60/68	PRL-PitNET	-	CAB, NS (3x), RT (50.4 Gy), PAS, TMZ (6 cycles)	IPI 1 mg/kg + NIVO 3 mg/kg 3/3 weeks	2	Progression after 2 cycles	Progression after 1 cycle	13/12 †
Duhamel et al. [39], 2020 *J Pers Med* (PMID: 32823651)	F	42/60	ACTH-PitNET (metastatic)	-	NS (3x), RT (50 + 25 + 45 Gy), TMZ (10 + 3 cycles), PAS, CAB, hydroxyurea	IPI 1 mg/kg + NIVO 3 mg/kg 3/3 weeks NIVO 3 mg/kg 2/2 weeks	5 21	Dissociated response with tumour growth and partial response of metastasis, followed by new metastasis	Partial response, followed by progression	14 †
Lamb et al. [40], 2020 *Front Endocrinol* (PMID: 33312158)	F	72	NF-PitNET (metastatic)	-	NS (3x), RT, RT metastasis, MS,TMZ (3cycles)	IPI 3 mg/kg + NIVO 1 mg/kg thrice weekly NIVO 3 mg/kg thrice weekly IPI 3 mg/kg + NIVO 1 mg/kg thrice weekly	2 17 4	Partial response, followed by progression Progression	n/a n/a	23/3
Majd et al. [41], 2020 *J Immunother Cancer* (PMID: 33427689)	M	Mid 30s	ACTH-PitNET (metastatic)	-	NS (3x), RT, RT metastasis, BA, TMZ (16 + 8 cycles), CAPTEM (1 + 4 cycles), MS, FGFR inhibitor (2 cycles), CCNU + BVZ (1 cycle)	Pembrolizumab 200 mg	29	Partial response	Complete response	42/22
Majd et al. [41], 2020 *J Immunother Cancer* (PMID: 33427689)	F	Early 20s	ACTH-PitNET (metastatic)	-	NS (2x), RT, BA, PAS, TMZ (7 cycles), CAPTEM (7 cycles)	Pembrolizumab 200 mg	15	Partial response	Immediate progression followed by partial response	12
Majd et al. [41], 2020 *J Immunother Cancer* (PMID: 33427689)	M	Late teens	NF-PitNET (metastatic)	-	NS (4x), RT, RT metastasis, TMZ (12 + 7 + 2 cycles), IDO1 inhibitor (11 cycles)	Pembrolizumab 200 mg	6	Stable	n/a	4
Majd et al. [41], 2020 *J Immunother Cancer* (PMID: 33427689)	F	Early 50s	PRL-PitNET (metastatic)	-	NS, RT, RT metastasis, CAB, Cisplatin/Etoposide, TMZ (12 + 2 cycles), CAPTEM (2 cycles)	Pembrolizumab 200 mg	6	Progression	Progression	4 †
Sol et al. [42], 2021 *Eur J Endocrinol* (PMID: 33112279)	M	41/48	ACTH-PitNET (metastatic)	NA	NS (2x), RT (2x), KET PAS, CAB, BA, TMZ (3 + 9 cycles)	IPI 3 mg/kg + NIVO 1 mg/kg 3/3 weeks NIVO 240 mg 2/2 weeks	4	Stable	Partial response	12
Burman et al. [35], 2022 *Eur J Endocrinol* (PMID: 36018781)	NA	NA	ACTH-PitNET	+	NA	NA	NA	Progression	NA	NA
Burman et al. [35], 2022 *Eur J Endocrinol* (PMID: 36018781)	NA	NA	ACTH-PitNET (metastatic)	NA	NA	NA	NA	Progression	NA	NA
Burman et al. [35], 2022 *Eur J Endocrinol* (PMID: 36018781)	NA	NA	ACTH-PitNET (metastatic)	NA	NA	NA	NA	Progression	NA	NA
Ilie et al. [4], 2022 *Endocr Relat Cancer * (PMID: 35521777)	M	55/66	ACTH-PitNET *	NA	NS (2x), RT (30 + 15 Gy), TMZ (23 + 8 + 12 cycles), BA	IPI 1 mg/kg + NIVO 3 mg/kg 3/3 weeks	4	Progression after 3 cycles	Progression after 4 cycles	14/12
Ilie et al. [4], 2022 *Endocr Relat Cancer * (PMID: 35521777)	M	51/73	NF- PitNET	+	NS (5x), RT (15 + 45 Gy), CAB, TMZ (3 cycles)	IPI 3 mg/kg + NIVO 1 mg/kg 3/3 weeks NIVO 3 mg/kg	5 1	Stable, followed by tumour growth	n/a	8/3 †
Ilie et al. [4], 2022 *Endocr Relat Cancer * (PMID: 35521777)	F	67/78	PRL-PitNET	-	NS (2x), CAB, TMZ (2x), PAS, TMZ + BVZ	IPI 1 mg/kg + NIVO 3 mg/kg 3/3 weeks	4	Stable, followed by tumour growth	Progression after 2 cycles	13/11
Ilie et al. [4], 2022 *Endocr Relat Cancer * (PMID: 35521777)	F	63/72	ACTH-PitNET	+	NS, RT (30 Gy), CAB, PAS, quinagolide, TMZ (17 cycles), BA	IPI 1 mg/kg + NIVO 3 mg/kg 3/3 weeks	5	Stable, followed by tumour growth	Progression after 2 cycles	11/8
Ilie et al. [4], 2022 *Endocr Relat Cancer * (PMID: 35521777)	M	39/44	ACTH-PitNET **	-	NS (2x),RT (54 Gy), TMZ (11 cycles)	NIVO 480 mg 4/4 weeks IPI 1 mg/kg 3/3 weeks	5 3	Stable, followed by disease progression	n/a	20/12
Ilie et al. [4], 2022 *Endocr Relat Cancer * (PMID: 35521777)	F	13/31	ACTH-PitNET	-	NS, RT (25 Gy), TMZ (12 + 7 cycles)	IPI 1 mg/kg + NIVO 3 mg/kg 3/3 weeks NIVO 3 mg/kg 2/2 weeks	4 25	Stable disease	NA	15
Ilie et al. [4], 2022 *Endocr Relat Cancer * (PMID: 35521777)	M	62/75	PRL-PitNET	+	CAB, NS (3x), RT (54 Gy), TMZ (7 cycles)	IPI 1 mg/kg + NIVO 3 mg/kg 3/3 weeks NIVO 480 mg 4/4 weeks	4 3	Stable, followed by tumour growth	Partial response followed by progression	13/3
Ilie et al. [4], 2022 *Endocr Relat Cancer * (PMID: 35521777)	M	35/43	ACTH-PitNET (metastatic)	-	NS (2x), RT (50.4 Gy), TMZ (9 cycles), PAS, everolimus, sunitinib	IPI 1 mg/kg + NIVO 3 mg/kg 3/3 weeks	4	Progression	NA	11/9
Ilie et al. [4], 2022 *Endocr Relat Cancer * (PMID: 35521777)	F	41/54	ACTH-PitNET	-	NS (3x), RT (50 Gy), CAB, PAS, BA, TMZ (21 + 6 cycles)	NIVO 240 mg 2/2 weeks IPI 1 mg/kg 3/3 weeks	4 4	Stable, followed by disease progression	Stable, followed by disease progression	12/7
Ilie et al. [4], 2022 *Endocr Relat Cancer * (PMID: 35521777)	M	26/39	PRL-PitNET (metastatic)	NA	CAB, NS (2x), MS, RT (54 Gy), RT metastasis (2x), TMZ (12 + 31 + 3 cycles), PAS, BVZ (7 + 2 cycles)	IPI 1 mg/kg + NIVO 3 mg/kg 3/3 weeks NIVO 3 mg/kg 2/2 weeks IPI 3 mg/kg 3/3 weeks	6 3 1	Stable, followed by disease progression	Partial response followed by progression	6
Ilie et al. [4], 2022 *Endocr Relat Cancer * (PMID: 35521777)	M	29/38	ACTH-PitNET (metastatic)	-	NS (3x), RT (54 + 15 Gy), BA, TMZ (3 + 15 cycles), BVZ (5 cycles)	IPI 1 mg/kg + NIVO 3 mg/kg 3/3 weeks NIVO 3 mg/kg 3/3 weeks	4 3	Partial response	Complete response	7
Ilie et al. [4], 2022 *Endocr Relat Cancer * (PMID: 35521777)	M	44/52	ACTH-PitNET (metastatic)	-	NS (2x), PAS, RT (50 + 24 Gy), RT metastasis (3x), CAB, BA, radiofrequency ablation of metastasis, TMZ (4 cycles)	IPI 1 mg/kg + NIVO 3 mg/kg 3/3 weeks NIVO 3 mg/kg 3/3 weeks	4 4	Dissociated response with partial response of tumour growth and progression of metastasis	NA	5
Feola et al. [11], 2022 *Cancers* (PMID: 36077631)	M	57	NF-PitNET (metastatic)	+	NS (3x), RT (2x), RT metastasis, TMZ (5 cycles)	Pembrolizumab 200 mg 21/21 days	>9	Partial response	n/a	12
Shah et al. [43], 2022 *Neurosurgery* (PMID: 35544035)	M	57	ACTH-PitNET	NA	NS, RT, TMZ (3 cycles)	IPI 3 mg/kg + NIVO 1 mg/kg 3/3 weeks NIVO 480 mg 4/4 weeks	4 10	Complete	Complete	15/7
Goichot et al. [36], 2023 *Clin Endocrinol* (PMID: 34845727)	M	41/54	PRL-PitNET (metastatic)	+	CAB, NS (2x), MS (2x), RT (50.4 + 50.4 + 37.5 Gy), TMZ (43 cycles), RT metastasis (4x)	IPI 3 mg/kg + NIVO 1 mg/kg 3/3 weeks NIVO 1 mg/kg 2/2 weeks	4 48	Partial response	Complete response	32/2
Medina et al. [15], 2023 Front Endocrinol (PMID: 37529607)	M	56/61	PRL-PitNET	+	NS (2x), RT (30 Gy), TMZ (>2 cycles),PZP	Pembrolizumab	NA	Progression	NA	3

*: initially silent; **: initially functioning PitNET; †: deceased when reported. ACTH: adrenocorticotropic hormone; BA: bilateral adrenalectomy; BVZ: bevacizumab; CAB: cabergoline; CCNU: lomustine; CAPTEM: capecitabine + temozolomide; FGFR: fibroblast growth factor receptor; IDO1: Indoleamine 2,3-dioxygenase 1; IPI: ipilimumab; KET: ketoconazole; MET: metyrapone; MIF: mifepristone; MS: metastasis surgery; NF-PitNET: non-functioning pituitary neuroendocrine tumour; NIVO: nivolumab; NA: not available; n/a: not applicable; NS: neurosurgery; PAS: pasireotide; PD-1: programmed cell death protein 1; PD-L1 status +: programmed cell death ligand-1 positive expression; PD-L1 status -: programmed cell death ligand-1 negative expression; PRL: prolactin; PRRT: peptide receptor radionuclide therapy; PZP: pazopanib; RT: radiotherapy; TMZ: temozolomide.

### 3.2. Anti-PD-1 Drugs and Posology in Aggressive or Metastatic PitNETs

Twenty-five out of the twenty-nine published PitNET cases reported data on ICI choice and posology. Nivolumab was used in dual therapy with ipilimumab in 17 cases, and in 12 of these, was followed by maintenance therapy with nivolumab (Table 1). In two ACTH-secreting PitNET cases, a sequential approach with nivolumab followed by ipilimumab was used (Table 1). Different nivolumab posologies have been used, including 1 mg/kg every 3 weeks, 3 mg/kg every 3 weeks, 240 mg every 2 weeks, and 480 mg every 4 weeks [4,36,37,39,40,42,43]. Pembrolizumab (200 mg) was used in monotherapy in six cases. One case was a non-metastatic PitNET, which was initially silent but became an ACTH-secreting PitNET during follow-up, and five were metastatic (two NF-PitNET, two ACTH-secreting, and one prolactinoma) [11,38,41]. Pembrolizumab was also used in addition to pazopanib in one case [15]. Positive PD-L1 expression was reported in four nivolumab-treated PitNETs and in two cases treated with pembrolizumab [4,11,15,36].

### 3.3. Radiological Response to Anti-PD-1 Drugs

The radiological response to anti-PD-1 drugs varied across the 29 PitNET patients (Table 2). The best radiological response was complete in one case (3.4%), partial in seven cases (24.1%), and stable in ten cases (34.5%). Nine patients (31%) had progressive disease upon anti-PD-1 treatment and there was a dissociated response in two PitNETs after the use of PD-1 inhibitors (*n* = 2, 6.9%) (Figure 1c).

**Figure 2:**
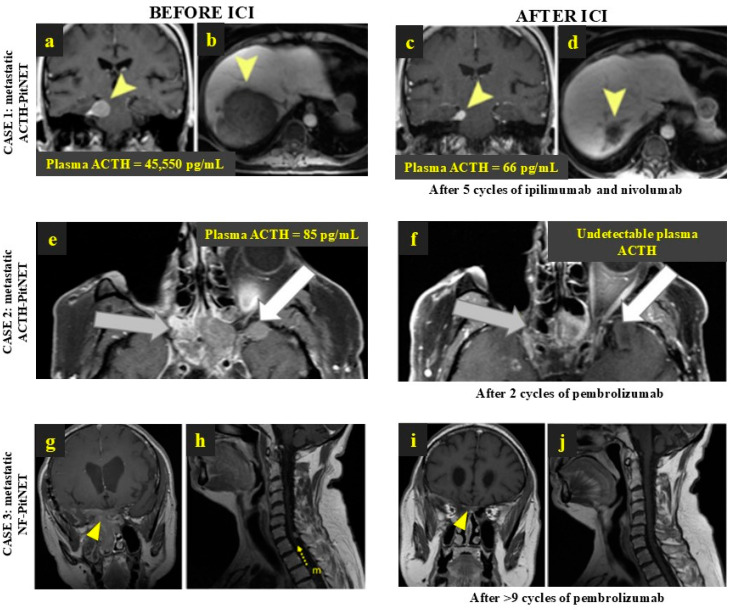
Remarkable response to anti-PD-1 treatment in 3 patients with metastatic PitNETs. CASE 1: Metastatic ACTH-PitNET with radiological and biochemical response to ipilimumab and nivolumab—brain and liver MRI before (**a**,**b**) and after ICI treatment (**c**,**d**). The yellow arrowheads point to the main tumour metastasis in the central nervous system and in the liver before and after ICI treatment. (Lin et al. 2018 [37], J Clin Endocrinol Metab (PMID: 30085142)). CASE 2: Metastatic ACTH-PitNET with radiological and biochemical response to pembrolizumab—MRI before (**e**) and after ICI treatment (**f**). The grey arrows point to tumour extension to the sphenoid sinus and posterior ethmoid air cells; white arrows indicate the tumour extension to the anterior and inferior aspects of the left temporal lobe. (Majd et al. 2020 [41], J Immunother Cancer (PMID: 33427689).) CASE 3: Metastatic NF-PitNET with radiological response to pembrolizumab, with >70% shrinkage in the main tumour and involution of spinal metastasis—MRI before (**g**,**h**) and after 1 year of ICI treatment (**i**,**j**). The yellow arrowheads show the primary PitNET before and after treatment, where a remarkable shrinkage of the tumour is visible; the yellow arrow with “m” letter depicts the spinal metastasis before ICI treatment, which involved following the treatment with pembrolizumab. (Feola et al. 2022 [11], Cancers (PMID: 36077631)). ACTH: adrenocorticotropic hormone; ACTH-PitNET: adrenocorticotropic hormone-secreting PitNET; FGFR: fibroblast growth factor receptor; ICI: immune checkpoint inhibitors; MRI: magnetic resonance imaging; NF-PitNET: non-functioning pituitary neuroendocrine tumour; PitNET: pituitary neuroendocrine tumour.

Among ACTH-secreting PitNETs, the radiological benefit after using ICIs was seen in 50% of the metastatic PitNETs (five of ten cases), as well as in 62.5% of the non-metastatic PitNETs (five of eight cases) (Table 3). A partial response following ICI was achieved in four metastatic ACTH-secreting PitNETs (two cases are shown in Figure 2a–f) while the other metastatic cases showed either stable disease (*n* = 1), progressive disease despite treatment (*n* = 4), or a dissociated response (*n* = 1). In non-metastatic ACTH-secreting PitNETs, four cases achieved stable disease, one case had complete involution, and three cases did not respond. The only case in which an outstanding complete response was reported concerned a male with a non-metastatic ACTH-secreting PitNET treated with surgery, radiotherapy, and three cycles of temozolomide prior to the dual therapy with ipilimumab and nivolumab, followed by 10 cycles of nivolumab [43].

Regarding prolactin-secreting PitNETs, the best radiological response in non-metastatic cases was stable disease in two out of four cases while the three metastatic cases responded differently: there was one case with a marked partial response accompanied by complete hormone control whereas stable disease followed by progression and the absence of a response were observed in the other two cases [4,36,41] (Table 3).

Among the seven PD-L1-positive PitNETs included, two were metastatic and five showed a radiological benefit after using ICIs: the PD-L1-positive metastatic NF-PitNET showed a partial but nearly complete local response (Figure 2g–j) while a remarkable response to ipilimumab and nivolumab was reported in the metastatic prolactin-secreting PitNET, which had previously been treated with cabergoline, neurosurgery, radiotherapy, and 43 cycles of temozolomide [11,36]. The other non-metastatic positive PD-L1 cases showed stable disease (*n* = 3) and progression in two cases [4,15,35].

Overall, in this series of 29 patients with aggressive or metastatic PitNETs who received ICI treatment, a positive radiological effect (i.e., partial or complete radiological response, or stable disease) was observed in 18 out of 29 cases (62.1%) (Table 2).

### 3.4. Biochemical Response to Anti-PD-1 Drugs

Of the 29 PitNET patients reported, 25 had functioning tumours and biochemical response data were available in 17 cases (Table 4). Positive biochemical response to ICIs was reported in eleven out of seventeen cases (64.7%); eight were ACTH-secreting PitNETs and three were prolactinomas. The best response was considered as complete in 23.5% of cases (*n* = 4), partial in 35.3% of cases (*n* = 6), and stable in one case (Figure 1d). Of six PitNETs that progressed despite ICI treatment, four received ipilimumab and nivolumab and two pembrolizumab. Complete biochemical response was achieved in two metastatic PitNETs; both received dual therapy with ipilimumab and nivolumab and additional monotherapy with nivolumab (long-term for forty-eight cycles and short-term for three cycles) [13,36]. A remarkable biochemical response to ipilimumab and nivolumab [37] and to pembrolizumab [41] is shown in Figure 2a–f. Among the four functioning PitNETs with PD-L1-positive expression, three had biochemical response data available: (i) a complete response to ipilimumab and nivolumab was documented in the metastatic prolactinoma, (ii) the non-metastatic prolactinoma showed a transient response to ipilimumab and nivolumab before further progression, and (iii) the ACTH-secreting PitNET did not respond to ipilimumab and nivolumab [13,36].

### 3.5. Survival Data

The median survival after commencing ICI treatment was 13 months, with a maximum of 42 months in two metastatic ACTH-secreting PitNET patients who underwent extensive treatments before starting 29 cycles of pembrolizumab or dual therapy with ipilimumab and nivolumab for 13 cycles [14,41].

Four patients became deceased despite treatment with ICIs: (i) a male with an aggressive non-metastatic prolactinoma whose disease progressed despite dual therapy with ipilimumab ad nivolumab; (ii) a female with a metastatic ACTH-secreting PitNET treated with ipilimumab and nivolumab, followed by maintenance therapy with nivolumab, who had a dissociated radiological response with the local progression of the primary tumour despite the involution of metastasis, followed by disease progression; (iii) a female with a metastatic prolactinoma that did not respond to pembrolizumab; and (iv) a male with a NF-PitNET with PD-L1-positive expression, who was treated with ipilimumab and nivolumab, who initially showed a stable disease followed by local growth [4,39,41].

## 4. Discussion

Isolated case reports and small series of PitNET patients treated with PD-1 inhibitors, reviewed here, support a potential role for PD-1 inhibitors in the management algorithm of aggressive or metastatic PitNETs, particularly when other therapeutic options have failed. In the cohort of 29 aggressive or metastatic PitNETs treated with anti-PD-1 drugs after prior therapies, we found that an overall beneficial radiological response was present in 62.1% of cases (3.4% complete response, 24.1% partial response, 34.5% stable disease) while an overall positive biochemical response was achieved in up to 64.7% of functioning PitNETs.

The experimental use of anti-PD-1 drugs thus far highlights a promising role for immunotherapy in advanced PitNETs as a salvage approach as such cases are often very aggressive and refractory to other treatments. Most of the published cases had several lines of therapy, including multiple surgeries, radiotherapy, temozolomide, and/or other experimental drugs, which rendered partial response or stable disease rates after ICI treatment of 24% and 35% as quite relevant in this setting. In fact, we found a clear clinical benefit in more than 60% of cases, with response rates in more than 25%, in a setting of patients who had few other treatment alternatives. Our pooled results regarding the efficacy of ICI treatment in patients with aggressive or metastatic PitNETs are encouraging, particularly when compared to other experimental therapies that have been used, such as those with bevacizumab or PRRT [3,44].

Our pooled analysis showed that a biochemical response may be achieved in over 60% of functioning PitNETs, with complete and partial biochemical responses occurring in 23.5% and 35.3% of the ICI-treated functioning PitNETs, respectively. Hence, in addition to the radiological response associated with ICIs, reflecting their anti-proliferative effects, anti-PD-1/PD-L1 drugs have useful properties in controlling hormone hypersecretion, particularly in aggressive or metastatic ACTH- and PRL-secreting PitNETs, which is often accompanied by tumour mass reduction [4,36,37,41,43]. Interestingly, a complete biochemical response was reported in two metastatic ACTH-secreting PitNETs and in one non-metastatic ACTH-secreting PitNET, which occurred concomitantly with a radiological response in all three cases [4,41,43].

In our pooled cohort, we observed a marked heterogeneity in tumour responses to anti-PD-1 drugs, ranging from complete remission to rapid disease progression after using ICIs [37,38,43], and that could have been related to several factors. Firstly, there are several difficulties in assessing tumour responses to immunotherapy with the current radiological tools, such as the RECIST criteria, which may start already in defining which one better applies (and how to better apply it) to the field of PitNETs [11,45]. Secondly, several ICI treatment protocols have been used in patients with advanced PitNETs, including different numbers of cycles and different doses and drug combinations, which limits the interpretation of treatment outcomes [4,39,40]. Thirdly, the timing of the radiological response assessment, which is particularly relevant as an early radiological evaluation, may lead to a misinterpretation of the response and inadequate premature ICI discontinuation [11]. Fourthly, the incomplete understanding of the microenvironment of PitNETs (which may be key for the responses to ICIs), as well as the lack of biomarkers predicting good response to ICIs in patients with PitNETs (including the positive expression of PD-L1 in pituitary tumour cells [27]), may lead to an inadequate selection of patients to receive such treatments [34,46]. Thus, the definition of standardised ICI treatment and imaging follow-up protocols, as well as an adequate patient selection and personalised management approach, are crucial not only to maximise the effectiveness of ICIs in patients with aggressive or metastatic PitNETs but also to ensure an adequate assessment of their effectiveness and safety.

An initial good response to ICI may be followed by treatment escape and the progression of disease. This is well illustrated by the case of a silent metastatic lactotroph PitNET patient who received ipilimumab and nivolumab, and then maintenance therapy with nivolumab, with a partial response sustained for 8 months, a period after which the tumour progressed again despite ICI treatment [40]. This escaping phenomenon has also been previously observed in immunotherapy for other cancers [47].

Another key aspect to unravel is related to whether immunotherapy is more effective in isolation or combined with other treatment modalities such as radiotherapy or PRRT. In fact, immunotherapy and radiation-based treatments may be synergic or complementary, as well illustrated by a reported case of a multi-treated metastatic corticotroph tumour that responded to ipilimumab and nivolumab [37] but eventually escaped, requiring a new therapeutic approach consisting of four cycles of PRRT [14]. After PRRT, the disease stabilised and nivolumab was resumed, and there was a further remarkable tumour reduction (~60%) accompanied by a marked decrease in serum ACTH levels [14]. It is plausible that radiation-related cell lysis uncovers antigenic sites, triggers cytokine release, and/or triggers immunomodulation, which ultimately leads to an immunogenic phenotype and sensitises PitNETs to ICIs [3], hence augmenting the ICI efficacy in such cases, as already described for other cancers [48,49]. In rare conditions such as with advanced PitNETs, where large clinical trials are unavailable, the potential use of novel therapies is often firstly assessed by experimental use in a single or few cases. Although these case reports are extremely valuable, they are insufficient for providing solid evidence about drug efficacy or safety, and they are also subject to a number of biases, including selection and publication biases, which may lead to the over- or under-reporting of treatment effectiveness [50]. The efficacy and safety of new treatments, including of ICIs when applied to PitNETs, require validation in randomised clinical trials. Currently, there are two ongoing clinical trials investigating the use of ICIs for patients with progressive, aggressive, or metastatic PitNETs: one phase-II trial assessing nivolumab combined with ipilimumab (four cycles) followed by maintenance therapy with nivolumab (six cycles) in patients with unresectable or metastatic PitNETs (NCT04042753) and one phase-II trial testing nivolumab combined with ipilimumab (for up to seventeen cycles) in patients with rare tumours including metastatic PitNETs (NCT02834013) [34].

## 5. Conclusions

In summary, a favourable radiological response to treatment with ICIs, including anti-PD-1 drugs (pembrolizumab and nivolumab), may occur in more than half of the patients with aggressive or metastatic PitNETs refractory to other therapeutic modalities. Additionally, control of the pituitary hormone excess may occur in a substantial number of patients with functioning PitNETs. These positive results, which may be observed in a substantial number of cases, translate into clinical benefits for a subgroup of patients with very limited options and support a role for attempting immunotherapy with ICIs in aggressive or metastatic PitNETs, particularly when other conventional treatments have failed.

## Figures and Tables

**Table 2:** Radiological response to PD-1 inhibitors in a cohort of 29 published PitNET patients according to aggressive or metastatic behaviour.

Radiological Response to Anti-PD-1 Treatment	Total PitNETs, *n* (%) *n* = 29	Metastatic PitNETs, *n* *n* = 16	Aggressive PitNETs, *n* *n* = 13
Complete	1 (3.4)	0	1
Partial	7 (24.1)	7	0
Stable disease	10 (34.5)	3	7
Dissociated	2 (6.9)	2	n/a
Progression	9 (31)	4	5
**Positive radiological response, *n* (%)**	18/29 (62.1)	10/16 (62.5)	8/13 (61.5)

Complete radiological response was considered when there was total remission of the primary tumour, partial response when the tumour involuted but disease remained present, stable disease when there was no clear response or progression, dissociated response when there was an opposite effect in the primary tumour and metastasis, and progression when there was lack of response to treatment. A positive radiological effect was considered in the case of partial/complete radiological or stable disease response. PitNETs were considered metastatic or aggressive according to the presence or absence of metastasis, respectively. n/a: not applicable; PD-1: programmed cell death protein 1; PitNET: pituitary neuroendocrine tumour.

**Table 3:** Radiological response to PD-1 inhibitors in a cohort of 29 published PitNET patients according to hormonal subtypes.

	ACTH-PitNET			PRL-PitNET			NF-PitNET		
Radiological response to Anti-PD-1 Treatment	Total, *n* (%) *n* = 18	Metastatic, *n* *n* = 10	Aggressive, *n* *n* = 8	Total, *n* (%) *n* = 7	Metastatic, *n* *n* = 3	Aggressive, *n* *n* = 4	Total, *n* (%) *n* = 4	Metastatic, *n* *n* = 3	Aggressive, *n* *n* = 1
Complete	1 (5.5)	0	1	0 (0)	0	0	0	0	0
Partial	4 (22.2)	4	0	1 (14.3)	1	0	2	2	0
Stable disease	5 (27.8)	1	4	3 (42.9)	1	2	2	1	1
Dissociated	1 (5.5)	1	n/a	0 (0)	0	n/a	0	0	n/a
Progression	7 (38.9)	4	3	3 (42.9)	1	2	0	0	0
**Positive radiological response, *n* (%)**	10/18 (55.5)	5/10 (50)	5/8 (62.5)	4/7 (57.1)	2/3 (66.6)	2/4 (50)	4/4 (100)	3/3 (100)	1/1 (100)

Complete radiological response was considered when there was total remission of the primary tumour, partial response when the tumour involuted but disease remained present, stable disease when there was no clear response or progression, dissociated response when there was an opposite effect in the primary tumour and metastasis, and progression when there was lack of response to treatment. A positive radiological effect was considered in the case of partial/complete radiological or stable disease response. PitNETs were considered metastatic or aggressive according to the presence or absence of metastasis, respectively. ACTH: adrenocorticotropic hormone-secreting PitNET; NF: non-functioning PitNET; n/a: not applicable; PD-1: programmed cell death protein 1; PitNET: pituitary neuroendocrine tumour; PRL: prolactin-secreting PitNET.

**Table 4:** Biochemical response to anti-PD-1 drugs in a cohort of 17 functioning PitNETs.

Biochemical Response to Anti-PD-1 Treatment	Total F-PitNETs, *n* (%) *n* = 17	ACTH-PitNET, *n* *n* = 11	PRL-PitNETs, *n* *n* = 6
Complete	4 (23.5)	3	1
Partial	6 (35.3)	4	2
Stable disease	1 (5.9)	1	0
Progressive disease	6	3	3
**Positive biochemical response, *n* (%)**	11/17 (64.7)	8/11 (72.7)	3/6 (50)

Only 17 of the 25 functioning tumours had biochemical response data available. The best biochemical response in functioning PitNETs was described as complete response when normal hormonal levels were achieved after anti-PD-1 treatment, partial response when hormone levels lowered but remained in excess, and stable when hormone secretion did not increase nor decrease after treatment. A positive biochemical effect of PD-1 inhibitors was assumed when hormone levels remained stable or decreased after treatment. ACTH: adrenocorticotropic hormone-secreting PitNET; F-PitNET: functioning PitNET; PD-1: programmed cell death protein 1; PitNET: pituitary neuroendocrine tumour; PRL: prolactin-secreting PitNET.
